# Prenatal Diagnosis of Severe Fetal Hydronephrosis Due to Pyeloureteral Junction Obstruction

**DOI:** 10.7759/cureus.95025

**Published:** 2025-10-21

**Authors:** Khadeeja Idrees, Roaa Elhaj, Douha Abdalla, Ahmed Mahmoud

**Affiliations:** 1 Obstetrics and Gynaecology, Glangwili General Hospital, Carmarthen, GBR; 2 Obstetrics and Gynaecology, South West Acute Hospital, Enniskillen, GBR

**Keywords:** hydronephrosis, neonatal, prenatal diagnosis, pyelo-ureteral junction, stenosis, ultrasound

## Abstract

Neonatal hydronephrosis is a common urinary tract anomaly often detected antenatally through routine fetal ultrasonography, requiring postnatal evaluation to determine its cause, severity, and need for intervention. We report the case of an infant diagnosed with isolated unilateral hydronephrosis shortly after birth, with serial imaging confirming persistent severe pelvicalyceal dilatation without contralateral involvement. Due to progressive obstruction and impaired drainage, the patient underwent unilateral pyeloplasty at six months of age. The postoperative course was uneventful, and follow-up imaging demonstrated excellent resolution of hydronephrosis with preserved renal function. This case underscores the importance of systematic postnatal evaluation of antenatal hydronephrosis, as early detection and monitoring enable timely intervention when necessary, thereby minimizing the risk of renal damage and optimizing long-term outcomes.

## Introduction

Fetal hydronephrosis is a common and readily identifiable condition on prenatal ultrasound, occurring in approximately 1-2% of pregnancies, with a higher incidence in males, where it is about twice as frequent as in females [1]. It presents bilaterally in 20-40% of cases and can be detected as early as 12-14 weeks of gestation [1]. However, ultrasounds performed before 18-24 weeks may miss clinically significant disease [2]. Studies extending into the third trimester have shown that the prenatal progression of hydronephrosis can help predict postnatal outcomes [2].

The primary objective of prenatal management is to identify cases of antenatal hydronephrosis that require postnatal assessment, timely referral to a pediatric urologist, and, when necessary, intervention [3]. To aid in diagnosis and risk stratification, the Society for Fetal Urology (SFU) established a grading system based on the degree of pelvic dilation, the number of visible calyces, and the extent of renal parenchymal thinning. In general, the severity of hydronephrosis correlates with the risk of underlying renal abnormalities. A meta-analysis involving 1,308 cases of antenatal hydronephrosis reported the likelihood of an abnormal postnatal outcome to be 88.3% for severe cases, 45.1% for moderate cases, and 11.9% for mild cases [3].

## Case presentation

**Figure 1:**
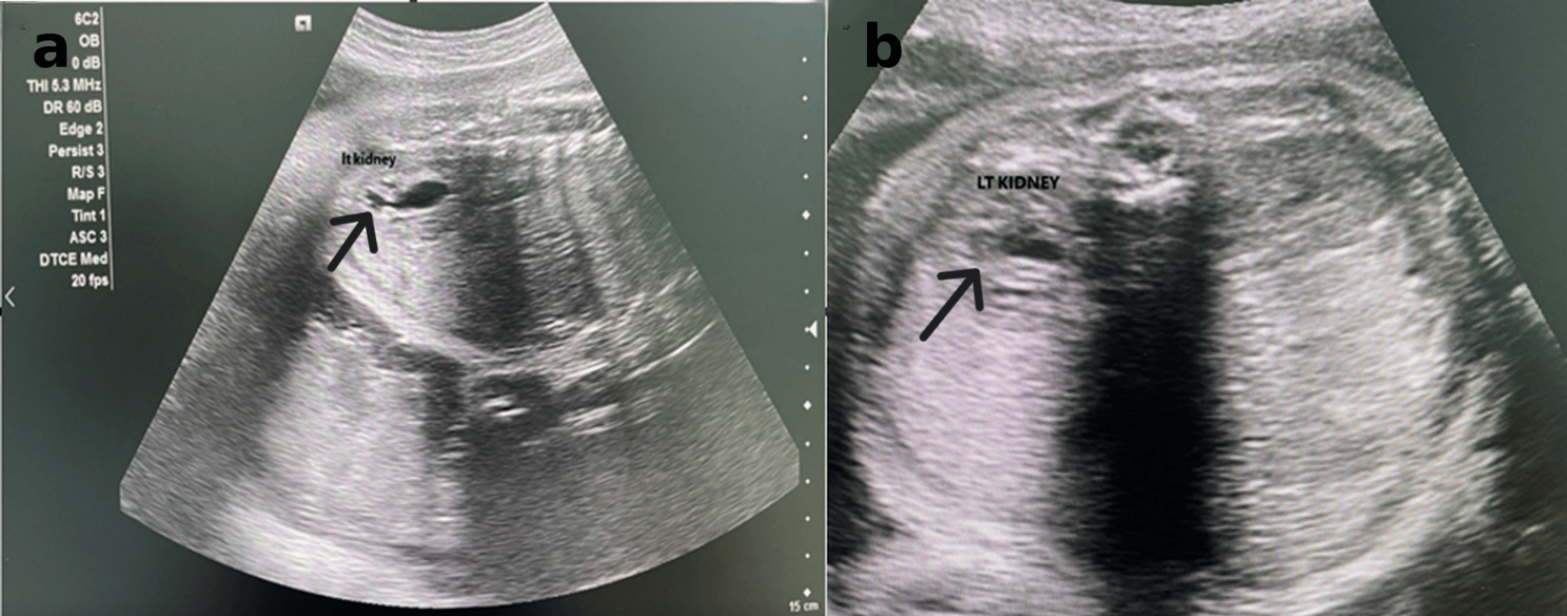
Neonatal scan of left kidney showing mild hydronephrosis; (a) sagittal view, (b) transection view.

A 37-year-old G4P3 woman was referred to our fetal medicine unit following a routine 20-week anomaly scan, which revealed a male fetus with isolated left-sided hydronephrosis. The case was referred for further evaluation and biweekly follow-up. Serial ultrasounds confirmed progressive dilatation of the left renal pelvis and calyces, while fetal biometry remained appropriate for gestational age and amniotic fluid volume was consistently normal. The urinary bladder appeared normal in size, shape, wall thickness, and voiding pattern. The left ureter was not visualized (Figure 1). Based on these findings, and after multidisciplinary discussion with pediatric urology specialists, a diagnosis of left-sided pyeloureteral junction (PUJ) obstruction with impaired urinary outflow was made.

The infant was delivered via spontaneous vaginal delivery at 39 weeks of gestation without complications. Postnatal evaluation was initiated promptly by the pediatric urology team, and antibiotic prophylaxis was commenced. On day 4 of life, renal ultrasound revealed only mild bilateral calyceal-pelvic dilatation, with a left renal pelvis anteroposterior (AP) diameter of 5 mm, without evidence of ureteral dilatation or parenchymal thinning. A voiding cystourethrogram (VCUG) was normal, and antibiotic prophylaxis was subsequently discontinued. The neonate was discharged in good condition.

**Figure 2:**
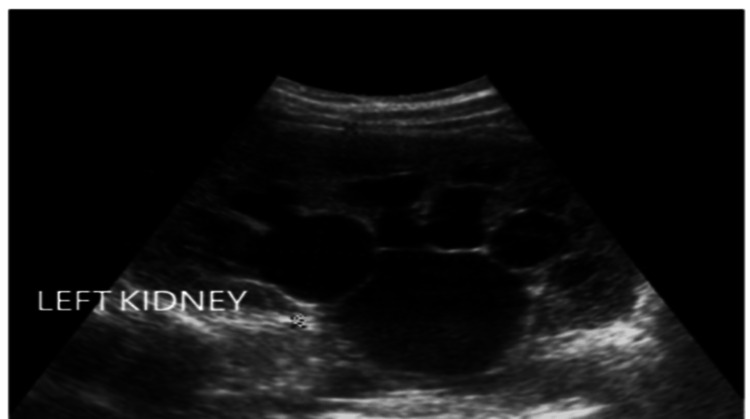
Ultrasound scan at two-three months showing significant left kidney hydronephrosis

At three months of age, follow-up ultrasound revealed enlargement of the left kidney, significant calyceal and renal pelvic dilatation, thinning of the renal parenchyma, and evidence of extra-renal pelvic expansion-all consistent with worsening obstruction. No ureteral dilatation was observed, and the findings persisted post-micturition (Figure 2).

By four months of age, further deterioration was noted with ultrasound findings indicating severe (Grade III) left hydronephrosis. Magnetic resonance urography (MRU) confirmed the diagnosis of left-sided PUJ obstruction. Surgical intervention was indicated, and the infant underwent a successful left pyeloplasty. The postoperative course was uncomplicated, and the internal stent was removed 15 days after surgery. Histological examination of the resected PUJ segment revealed subepithelial fibrosis, confirming the chronic obstructive nature of the lesion.

The infant recovered well and showed progressive improvement in renal drainage on follow-up imaging.

## Discussion

As highlighted in this report, early detection of PUJ obstruction through prenatal ultrasound is crucial. Studies indicate that isolated unilateral hydronephrosis is often identified during routine prenatal screening, with varying degrees of severity observed. Regular follow-up is essential to monitor the progression of hydronephrosis and to assess the need for intervention [4].

Postnatal imaging, including renal ultrasound and VCUG, is the standard approach for evaluating renal function and ruling out other anomalies. In the present case, initial imaging demonstrated mild calyceal-pelvic dilatation without significant renal parenchymal alteration. This finding is consistent with reports suggesting that many cases of neonatal hydronephrosis resolve spontaneously or remain stable without surgical intervention [5].

Histopathological examination of the resected PUJ segment revealed subepithelial fibrosis, a common finding that reflects the chronic nature of the obstruction. This aligns with prior studies reporting similar histological changes in patients undergoing pyeloplasty for PUJ obstruction [6].

The postoperative course in this patient was favorable, with resolution of hydronephrosis and preservation of renal function. Long-term follow-up remains essential to ensure continued renal health, as studies indicate that while many cases of neonatal hydronephrosis improve over time, some ultimately require surgical management.

## Conclusions

Isolated unilateral neonatal hydronephrosis due to PUJ obstruction is a common but potentially progressive condition. Early diagnosis, structured follow-up, and timely surgical intervention in appropriate cases are essential to preserve renal function and optimize outcomes. Further research into the molecular and genetic mechanisms underlying PUJ obstruction may enhance risk stratification and improve management in the future.
